# Non-Invasive Drug Delivery across the Blood–Brain Barrier: A Prospective Analysis

**DOI:** 10.3390/pharmaceutics15112599

**Published:** 2023-11-07

**Authors:** Sarfaraz K. Niazi

**Affiliations:** College of Pharmacy, University of Illinois, Chicago, IL 60612, USA; sniazi3@uic.edu

**Keywords:** neurodegenerative disorders, blood–brain barrier, non-invasive delivery, device-related delivery, Alzheimer’s disease, Parkinson’s disease, ALS, Down syndrome

## Abstract

Non-invasive drug delivery across the blood–brain barrier (BBB) represents a significant advancement in treating neurological diseases. The BBB is a tightly packed layer of endothelial cells that shields the brain from harmful substances in the blood, allowing necessary nutrients to pass through. It is a highly selective barrier, which poses a challenge to delivering therapeutic agents into the brain. Several non-invasive procedures and devices have been developed or are currently being investigated to enhance drug delivery across the BBB. This paper presents a review and a prospective analysis of the art and science that address pharmacology, technology, delivery systems, regulatory approval, ethical concerns, and future possibilities.

## 1. Introduction

**Figure 1:**
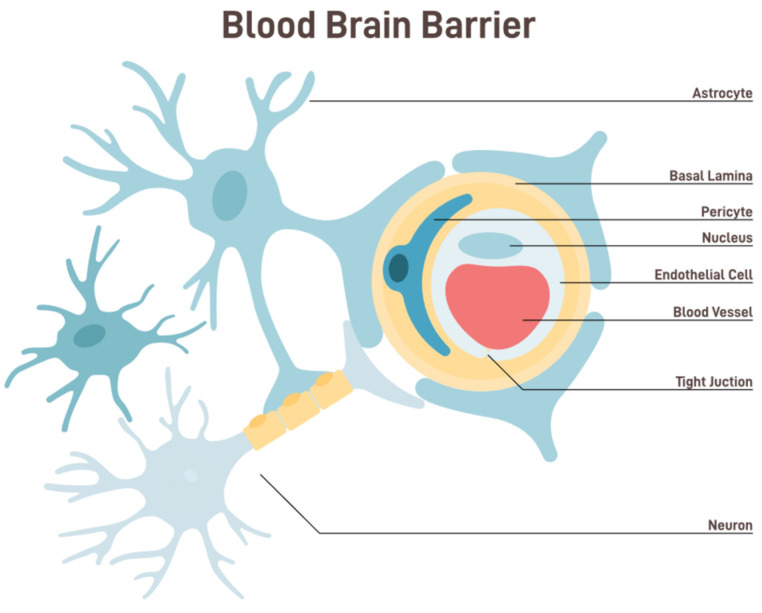
Anatomy and functional components of BBB [shutterstock_2229011587].

Evolutionarily, the brain is the body’s control center, responsible for everything from essential autonomic functions like heartbeat and respiration to complex cognitive tasks and emotional processing [1]. The human brain, with its intricate architecture and countless functions, is undeniably one of the most sophisticated organs in the body [2]. Protecting and ensuring the optimal functioning of this organ is of paramount importance. The BBB is central to this protective mechanism, a physiological marvel that safeguards the neural environment from potential toxins and pathogens [3] (Figure 1). However, the very features that make the BBB an efficient protector make it a formidable neurotherapeutic obstacle [4].

The BBB is a semipermeable border that separates the circulating blood from the brain and extracellular fluid in the central nervous system (CNS) [5]. This barrier comprises endothelial cells lining the capillaries, which are closely packed together and sealed by tight junctions. These tight junctions restrict the passive diffusion of large or hydrophilic molecules into the CNS. Additionally, astrocyte foot processes wrap around the blood vessels, further fortifying this barrier and playing a pivotal role in its function [6]. The BBB, while allowing essential nutrients like glucose and amino acids to reach the brain, filters out potentially harmful substances from entering the neural environment. This selective permeability ensures that the brain remains relatively insulated from fluctuations in blood composition, thereby maintaining a stable internal environment [7].

This paper outlines the current and future approaches to enhance BBB penetration to treat multiple brain diseases, including tumors, NDs, physiological disbalance, and other continually discovered disorders, using non-invasive devices and associated methods to optimize the functionality of these devices. These supportive approaches may include regulating circadian rhythms, restoring the gut milieu, opening the transitory BBB, carrier-mediated drug delivery, nasal administration, and activating signaling pathways.

The BBB prevents substances from freely passing between the bloodstream, brain, and CNS. This selective and semi-permeable barrier is essential in developing and managing neurological disorders (NDs) that include Alzheimer’s disease (AD), Parkinson’s disease (PD), Huntingdon disease (HD), Amyotrophic lateral sclerosis (ALS), and multiple sclerosis (MS) and are characterized by the progressive loss of neurons that are associated with neurotoxic etiological substances in the brain and the surrounding organs.

Protecting the brain from toxins, pathogens, and other foreign substances is crucial for survival. Thus, the BBB has evolved to become a gatekeeper, ensuring that only substances beneficial or neutral to the brain’s function gain entry [8]. However, this protective shield also presents a significant challenge for medical science, particularly neurology and psychiatry [9]. Most drugs designed to target the brain—whether for the treatment of neurodegenerative diseases, psychiatric disorders, or brain tumors—cannot cross the BBB in therapeutically effective dosing [10], leading to numerous potential treatments failing in the clinical stages, not necessarily because the drugs are not efficacious, but because they cannot reach their intended site of action in the brain [11]. Thus, the BBB represents a double-edged sword. While it is indispensable for physiological well-being, it is also one of the most formidable obstacles in treating neurological diseases [12].

As the understanding of neurological and psychiatric disorders deepens, and with the advent of precision medicine, there is an escalating demand for treatments that can be tailored to individual patient needs [13]. Such treatments may necessitate frequent or prolonged drug administration. Apart from their inherent risks, the invasive methods become impractical in these contexts due to their invasive nature and the discomfort associated with repeated interventions.

Researchers and clinicians have long recognized this challenge [14]. Over the years, various strategies have been employed to overcome or bypass the BBB. Some of these methods are invasive, such as direct intracerebral injections, which, while effective, come with risks and complications [15]. As a result, the focus has increasingly shifted towards non-invasive approaches, aiming to safely enhance the delivery of therapeutic agents to the brain without compromising the integrity of the BBB [16].

The brain’s homeostasis is significantly influenced by its function. The BBB comprises diverse cell types and structures, such as brain endothelial cells, the basement membrane, tight junctions, astrocytes, and pericytes. These components collaborate harmoniously to establish a highly selective and tightly regulated barrier [17,18]. The BBB is vital in maintaining homeostasis inside the central CNS by serving as a critical interface between the peripheral circulatory system and the brain. This barrier employs a range of methods to fulfill its function. The BBB is a protective mechanism against the infiltration of detrimental chemicals into the brain.

Additionally, it plays a crucial role in regulating the equilibrium of ions, sustaining optimal levels of neurotransmitters, and eliminating metabolic waste. Nevertheless, as individuals age, there is a potential for the BBB to have a decline in its structural integrity [19]. Multiple studies have provided evidence for the significant involvement of the BBB in the development of numerous neurological disorders [20]. Furthermore, the BBB presents a substantial impediment to the administration of drugs in the context of neurodegenerative diseases [21]. Consequently, research about the BBB has exhibited both diversification and simultaneous advancement.

### Selective Mechanism

**Figure 2:**
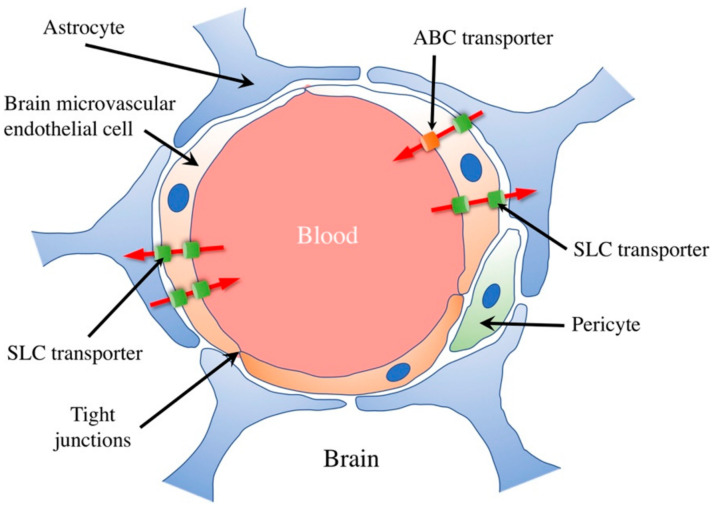
Schematic illustration of the blood–brain barrier and transporters. The BBB comprises brain microvascular endothelial cells, astrocytes, and pericytes. Their tight junction-mediated mutual binding restricts diffusion between brain microvascular endothelial cells. Numerous soluble carrier (SLC) transporters expressed by brain microvascular endothelial cells enable specific material passage across the blood–brain barrier, including nutrients like glucose, amino acids, peptides, and nucleotides. Furthermore, by releasing harmful compounds and medications into the bloodstream, ATP-binding cassette (ABC) transporters, expressed in cerebral microvascular endothelial cells, block their access to the brain. [Shutterstock_2229011587].

Given the complexity of the BBB, it is essential to understand its selectivity mechanisms. Efflux and influx transporters are pivotal in determining which molecules can enter or exit the brain [22] (Figure 2).

The physicochemical properties of drugs play a crucial role in their ability to cross the BBB. Lipophilicity, molecular weight, and charge are vital factors influencing BBB permeability. Lipophilic compounds, such as small, nonpolar molecules, diffuse more readily through the lipid-rich endothelial cell membranes. Drugs with lower molecular weights are generally favored for BBB penetration, as they can more efficiently navigate the narrow intercellular spaces. However, highly charged or polar compounds may face significant obstacles as the BBB restricts their passage. Efflux transporters, such as P-glycoprotein, actively pump out xenobiotics, further limiting drug access to the brain. Medicinal chemists often design compounds with favorable physicochemical properties, such as logP values, to enhance brain penetration and consider prodrug strategies or nanocarrier systems. Understanding these properties is critical for the development of drugs targeting neurological disorders, as it ensures their ability to reach their intended site of action in the CNS [23]. Historically, attempts to augment drug delivery to the brain focused on chemical modifications to therapeutic agents, enabling them to either permeate or be actively transported across the BBB [24]. However, these modifications often altered pharmacokinetics or diminished therapeutic efficacy, resulting in compromised treatment outcomes [25]. The realization that chemical modifications could only achieve limited success shifted the emphasis toward more direct, though invasive, delivery methods [26].

In addition to physicochemical properties, the transport of drugs across the blood-brain barrier is also influenced by specific transport mechanisms. Small lipophilic drugs can passively diffuse through the lipid bilayer of the endothelial cells, but for many drugs, active transport mechanisms are required for efficient entry into the brain. Several transporters and receptors at the BBB facilitate or restrict drug passage. For example, glucose transporters (GLUT1) allow glucose and certain related compounds to cross the BBB through facilitated diffusion. Similarly, amino acid transporters help transport essential amino acids into the brain.

**Figure 3:**
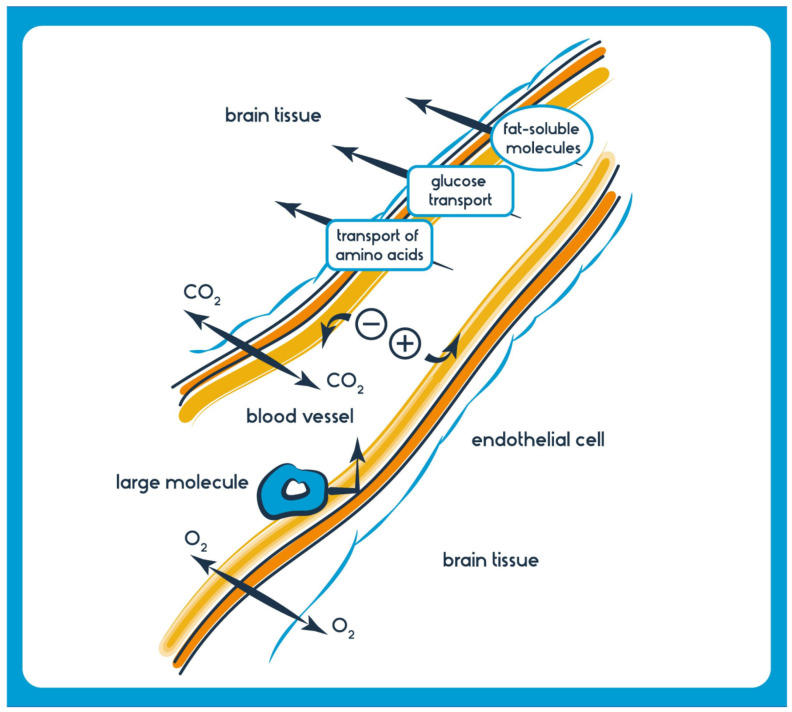
Beyond transporters, the physicochemical properties of drug molecules also determine their BBB permeability. [Shutterstock_555919957 Photo] Channels (one way from blood to brain): Small ions and water; Membrane transport (one way from blood to brain): Small lipophilic molecules such as oxygen, carbon dioxide, anesthetics, ethanol, nicotine, and caffeine; Carrier-Mediated Transport (soluble carriers) (one way or two way): Energy Transport System: glucose (GLUT-1), monocarboxylates, lactate, pyruvate (MCT1), creatinine (CrT), Amino acid transport system: large amino acids (LAT1), and Organic anion/cation transporters: OATP1A2, nucleosides; Receptor-mediated Transport (one way from blood to BBB to brain): Insulin, transferrin, leptin, IgG, TNF alpha; Adsorption-mediated Transcytosis System (one way from blood to BBB to brain): Histone, Albumin; Active Efflux Transporters (one-way from BBB to blood and brain): P-glycoprotein, BRCP, MRP 1,2,4,5. Several brain diseases, including neurodegenerative disorders like AD, PD, and ALS, as well as brain tumors and infections, often necessitate drugs that must effectively cross the BBB to exert their therapeutic effects [27]. Generally, lipophilic molecules with a molecular weight of less than 400–500 Da can traverse the BBB more efficiently [28]. However, many potent CNS-active drugs and biologics are too large or possess unsuitable properties, preventing their direct passage through the barrier.

On the other hand, efflux transporters like P-glycoprotein (encoded by the ABCB1 gene) actively pump out drugs from the brain back into the bloodstream, limiting their accumulation. Researchers often explore strategies to exploit these transporters for drug delivery, such as prodrugs that are substrates for specific transporters or the development of receptor-targeted delivery systems. Understanding the interplay between drug properties and transport mechanisms is vital for designing effective treatments for neurological diseases and ensuring optimal drug concentrations within the brain (Figure 3). 

The challenges for the optimal delivery of drugs to the brain underscore the urgent need for innovative strategies to deliver drugs across the BBB without compromising its integrity or function. As we look towards non-invasive device-mediated techniques, it is not just about bypassing the BBB but about maintaining the health and functionality of the CNS, ensuring that treatments are both practical and safe [29].

Beyond neurodegenerative diseases, non-invasive device-mediated techniques can potentially be employed in psychiatric disorders, rehabilitation, and even enhancing cognitive abilities [30]. Preliminary studies are exploring the role of transcranial magnetic stimulation in enhancing memory and cognitive functions in healthy and diseased brains [31].

Moreover, the BBB is not just a static barrier. Its permeability and function can be altered under pathological conditions. Diseases like stroke, traumatic brain injury, and certain infections can disrupt BBB integrity, which might allow for increased drug delivery but at the cost of potential harm from other circulating substances.

The potential for off-target effects, especially when breaching the BBB, has raised safety concerns. Unintended opening of the BBB or delivering therapeutics to non-targeted areas could lead to adverse outcomes [32]. In a clinical study assessing the effects of FUS on BBB disruption, a few patients exhibited temporary neurologic deficits, underscoring the need for meticulous planning and precision in application [33].

## 2. Optimizing Entry

A silver lining in this quest has been the discovery that the BBB, while protective, has specific “windows” or mechanisms that can be modulated for therapeutic benefit. For instance, specific peptides have been identified that can transiently open the BBB, allowing for drug delivery without causing lasting damage [34]. As researchers dive deeper into understanding these nuances, the dream of non-invasively and effectively delivering drugs to the brain seems increasingly tangible.

Non-drug measures to enhance BBB penetration include the use of focused ultrasound, which temporarily disrupts the barrier, allowing drugs to pass through [35]. Additionally, strategies such as nanoparticle-based drug delivery systems, which can encapsulate and protect drugs during transit across the BBB, are under investigation [36]. Nanocarriers can be functionalized with ligands targeting specific receptors at the BBB for enhanced transport [37].

Emerging technologies like intranasal drug delivery, which bypasses the BBB through the olfactory pathway, and implantable devices for direct drug administration into the brain are also being explored as potential solutions to overcome the BBB’s limitations [38,39]. These non-drug measures and innovative devices offer promising avenues to improve drug delivery to the brain in the treatment of various neurological disorders.

In addition to the non-drug measures and non-invasive devices, the use of carrier molecules, such as antibodies or peptides, that can specifically target receptors or transporters on BBB endothelial cells can facilitate the transport of therapeutic agents into the brain.

Convection-enhanced delivery (CED) is another technique that involves the direct infusion of drugs into brain tissue using surgically implanted catheters, bypassing the BBB and allowing for precise drug distribution [40].

Additionally, researchers are investigating the potential of nanoscale drug carriers like liposomes and exosomes, which can encapsulate drugs and transport them across the BBB [41]. Advances in nanotechnology and innovative drug delivery strategies hold promise in overcoming the challenges associated with BBB penetration, offering new avenues for effectively treating brain disorders.

Exosome-based drug delivery systems, leveraging the natural ability of exosomes to cross the BBB, have demonstrated potential in delivering neurotherapeutics.

In addition to the methods and approaches mentioned earlier, ongoing research efforts explore the potential of external devices and techniques to assist drug delivery across the BBB. One such technique is the use of focused ultrasound in combination with microbubbles. This non-invasive approach, known as focused ultrasound-mediated blood–brain barrier opening (FUS-BBBO), involves the application of focused ultrasound waves to the brain in the presence of microbubbles, temporarily disrupting the BBB and allowing drugs to pass through [42]. This method has shown promise in clinical trials for conditions like Alzheimer’s disease and brain tumors [43].

Additionally, implantable devices and catheters equipped with drug reservoirs or pumps can provide controlled and sustained drug delivery directly to the brain or cerebrospinal fluid (CNS). These devices can be programmed to release drugs at specific intervals, optimizing treatment efficacy while minimizing systemic side effects [44]. Furthermore, advancements in nanotechnology have led to the development of implantable, biodegradable, drug-eluting nanofibers that can release drugs over extended periods, offering a potential solution for chronic neurological conditions [45].

## 3. Device-Mediated Noninvasive Techniques

This shift towards non-invasive, device-mediated approaches is not just motivated by the limitations of traditional methods but also inspired by the potential these techniques have demonstrated, both in pre-clinical models and in some early-stage clinical trials. They herald a new era in neurotherapeutics, where treatments are effective, patient-centric, and tailored to the needs and comfort of individuals [46].

However, the dawn of the 21st century has witnessed rapid advancements in biomedical engineering, nanotechnology, and imaging modalities [47]. These advances have facilitated the development of device-mediated techniques that are minimally invasive or completely non-invasive, marking a seismic shift in the approach toward CNS drug delivery [48].

Recent years have seen a surge in interest and research into device-mediated techniques that could potentially surmount the BBB without requiring direct surgical intervention [49]. These techniques aim to open the BBB temporarily and safely or utilize specialized mechanisms to transport drugs. These technologies, including focused ultrasound, electromagnetic fields, and intranasal delivery, promise to revolutionize CNS drug delivery [50].

It is crucial to underline, however, that with the exhilaration of these breakthroughs comes the weighty responsibility of ensuring that these methods are safe. The BBB is a vital protective structure, and any strategy that seeks to circumvent or modulate its function must do so without compromising its long-term integrity or inducing unwanted side effects [51]. After all, these innovative approaches aim to improve patient outcomes and quality of life.

As these device-mediated techniques evolve and mature, they will be subjected to rigorous testing in pre-clinical settings and clinical trials. This will ensure their efficacy and safety profile, which is crucial for any therapeutic intervention targeting the delicate and intricate environment of the CNS.

In summary, while several methods exist to address the BBB’s drug delivery challenge, many drawbacks limit their therapeutic potential. The emergence of non-invasive device-mediated techniques represents a significant leap forward, potentially revolutionizing CNS drug delivery. As research progresses, the focus will undoubtedly shift towards refining these techniques, ensuring their safety, and expanding their therapeutic applicability [52].

To overcome the barriers to entry, a multitude of BBB in vivo and in vitro models have been established alongside innovative methodologies, which exhibit significant promise for conducting mechanistic investigations and advancing drug discovery efforts, such as the length of time a medication remains detectable in the body while administering pharmacological therapy to optimize the effectiveness of drug transportation across the BBB. The incorporation of novel technologies that effectively regulate the temporary permeability of the BBB and enable targeted drug delivery without invasive procedures is of utmost importance in enhancing the effectiveness, safety, and practicality of therapeutic approaches.

Non-invasive devices and techniques are gaining prominence as valuable tools for enhancing drug delivery across the BBB. One such approach is transcranial magnetic stimulation (TMS). TMS involves using magnetic fields to induce electrical currents in specific brain regions. Studies have shown that repeated TMS sessions can transiently increase BBB permeability, allowing for improved drug penetration into the brain [53].

Another non-invasive technique uses near-infrared spectroscopy (NIRS) and functional near-infrared spectroscopy (fNIRS) to monitor real-time BBB integrity. By assessing BBB disruption, researchers can optimize the timing of drug administration to maximize therapeutic benefits [54]. Furthermore, researchers are exploring non-invasive brain stimulation techniques, such as transcranial direct current stimulation (tDCS) and transcranial alternating current stimulation (tACS), to modulate BBB permeability. These methods can potentially enhance drug delivery without the need for invasive procedures [55].

Another non-invasive device that garnered attention is functional magnetic resonance imaging (fMRI) or magnetic resonance imaging (MRI) guided focused ultrasound. This technology combines the precision of MRI to visualize brain regions with the capability of focused ultrasound to disrupt the BBB temporarily. By using real-time MRI guidance, physicians can precisely target and monitor the delivery of drugs or other therapeutic agents, ensuring accurate and localized treatment [35]. Non-invasive devices and technologies like fMRI-guided focused ultrasound and wearable EEG devices represent exciting prospects for improving drug delivery to the brain while maintaining patient comfort and safety. These methods are still evolving but hold significant promise for enhancing the treatment of neurological diseases.

The evolution of wearable tech can allow continuous or periodic drug delivery, providing patients with more autonomy and ensuring sustained therapeutic levels [56]. A prototype wearable ultrasonic patch, capable of crossing the BBB and delivering drugs, showed promise in maintaining therapeutic drug levels in PD models [57]. Additionally, emerging technologies like wearable electroencephalogram (EEG) devices are being explored to modulate BBB permeability non-invasively. By using an EEG to guide the timing of drug administration or the application of other techniques, researchers aim to optimize drug delivery to the brain while minimizing side effects [58].

While current research predominantly focuses on neurodegenerative disorders and brain tumors, there is potential to extend these techniques to other conditions, like psychiatric disorders, autoimmune diseases, or metabolic conditions [59].

## 4. Brain Tumors

Brain cancer is characterized by the uncontrolled growth of cells in the brain, resulting in tumors that can be either benign or malignant. Several primary brain tumors exist, including gliomas, which arise from glial cells and encompass subtypes like astrocytomas and glioblastomas, with the latter being notably aggressive. Meningiomas originate from the meninges, while pituitary tumors are typically benign and develop in the pituitary gland. Medulloblastomas are more common in children and affect the cerebellum. Acoustic neuromas, originating from the cranial nerves’ Schwann cells, are generally benign but can impact hearing and balance. Diagnosis involves clinical evaluation, imaging studies, and, occasionally, a biopsy. Treatment is contingent on the tumor’s type, location, and stage and might include surgery, radiation therapy, chemotherapy, or newer modalities like targeted therapy and immunotherapy. However, the presence of the blood–brain barrier, the sensitive location of tumors, and resistance to treatment modalities complicate brain cancer treatment.

Molecular profiling constitutes a fundamental component of the 2021 World Health Organization (WHO) categorization of gliomas. The advancement of targeted therapy is currently constrained by various variables, including the existence of the BBB and challenges associated with tumor heterogeneity. However, significant progress has been achieved in developing the IDH1/2 inhibitor vorasidenib for treating IDH-mutant grade 2 gliomas. Additionally, the combination of dabrafenib and trametinib has shown promising results in treating gliomas with the BRAFV600E mutation. Furthermore, targeted medicines have been developed for certain groups of patients with fusions and H3K27M-altered diffuse midline gliomas [60].

Treatments for brain tumors like glioblastoma are limited by the inability of many chemotherapeutics to penetrate the BBB. Non-invasive techniques promise enhanced delivery of tumor-fighting drugs directly to the malignancy, potentially improving outcomes [35].

In a pioneering clinical trial, patients with recurrent glioblastoma received a combination of FUS and microbubbles before administering doxorubicin, a chemotherapy agent. The subsequent MRI scans showed increased drug concentrations in tumor regions, suggesting effective BBB disruption and targeted delivery [61]. Preliminary research showed the potential for focused ultrasound to modulate neural circuits associated with depression, opening avenues for non-drug treatments in psychiatric conditions [62].

A recent endeavor combined MRI-guided focused ultrasound with nanoparticles to ensure real-time visualization and precise drug delivery for treating tumors [35].

Convergence of Technologies: In the age of rapid technological advancements, the fusion of multiple technologies can further enhance the precision and efficiency of drug delivery. Imagine integrating real-time imaging with delivery devices to ensure pinpoint accuracy [63].

## 5. Neurodegenerative Disorders (NDs)

Neurological disorders affect millions globally, ranging from NDs to psychiatric conditions. These conditions lead to significant morbidity and have profound social, economic, and psychological repercussions [64]. Therefore, developing efficacious treatment strategies that can navigate the BBB’s complexities is paramount.

Neurodegenerative diseases, more particularly PD, AD, HD, ALS, and motor neuron disease, affect millions of people worldwide. NDs manifest as the progressive loss of functionality and eventual demise of nerve cells in the brain or peripheral nervous system. While specific treatments may provide relief for certain physical or mental symptoms commonly associated with neurodegenerative diseases, the current medical understanding does not support the notion that these treatments can effectively slow down the advancement of such diseases. Furthermore, it is important to note that no known cures for neurodegenerative disorders currently exist.

The probability of acquiring a neurodegenerative disease increases significantly as an individual advances in age. In the forthcoming decades, NDs have the potential to substantially impact a larger proportion of the American population, particularly as life expectancy continues to rise. It is imperative to enhance our comprehension of the etiology of neurodevelopmental disorders (NDs) and devise novel strategies for their treatment and prevention.

AD and PD are widely recognized as the prevailing neurodegenerative disorders. According to a report released by the Alzheimer’s Disease Association in 2022, it is estimated that up to 6.2 million individuals [65] in the United States may be affected by AD. According to the Parkinson’s Foundation, the number of individuals in the United States living with Parkinson’s disease is approaching one million [66].

AD is primarily distinguished by the degeneration of neurons, the accumulation of amyloid-beta plaques, and the development of hyperphosphorylated tau protein within neurons. Notably, the cytotoxic impact of amyloid beta-peptide (Abeta) is a significant factor in this disease [67].

AD is the accumulation of amyloid-β plaques, a hallmark of AD. Traditional drug delivery strategies have struggled to effectively delivering therapeutic agents across the BBB to target these plaques. Using focused ultrasound combined with microbubbles, research has demonstrated the potential to temporarily open the BBB and assist in the clearance of these plaques, showcasing potential therapeutic benefits [68]. A preclinical study involving mice genetically predisposed to develop AD symptoms revealed that after multiple FUS treatments, there was a notable reduction in amyloid-β plaques and improved cognitive function [69].

The phenotypic characteristic of Down syndrome (DS; trisomy 21) is now acknowledged to be associated with a significantly elevated risk for AD. The cumulative incidence of AD in individuals with DS increased to around 50% by the late 50s and reached 80% by the late 60s [70]. This demographic constitutes the most noteworthy cohort with an elevated susceptibility to AD due to a particular genotype. This situation is essential to public health and presents an ideal population for preventative trials.

The loss of dopaminergic neurons characterizes PD. Delivering neuroprotective or neurorestorative agents into the brain holds therapeutic promise. Techniques like FUS can facilitate the delivery of such agents, including genes or stem cells [71]. A study on a PD animal model demonstrated that after FUS-mediated BBB disruption, there was enhanced delivery and retention of neurotrophic factors, subsequently leading to improved motor functions in the treated animals [72]. A study combining focused ultrasound with patient-specific MRI data demonstrated more accurate targeting, leading to better therapeutic outcomes in PD patients [73].

The expression levels of Wnt3, Wnt4, FZD2, FZD8, Wnt2b, Wnt5a, FZD3, LRP5, and sFRP3 are elevated in the human spinal cord tissue of patients diagnosed with ALS, wherein there is an increase in the population of astrocytes expressing the FZD2 receptor in the transitional region between the grey and white matter at the ventral horn. The potential involvement of the Wnt family of proteins, particularly FZD2 and Wnt5a, in the pathogenesis of ALS is under investigation [74].

It is widely acknowledged among scientists that the interaction between an individual’s genetic makeup and their environmental factors significantly contributes to their susceptibility to neurodegenerative disorders. As an illustration, an individual may possess a genetic predisposition that renders them more vulnerable to PD. Yet, the impact of their environmental exposures can influence the disease’s timing, severity, and manifestation.

Post-stroke treatments can benefit from timely and targeted delivery of neuroprotective agents or stem cells. Non-invasive techniques can enhance the penetration of these therapeutic agents, potentially reducing brain damage and promoting recovery [75].

## 6. Current Techniques in Drug Delivery across the BBB

**Figure 4:**
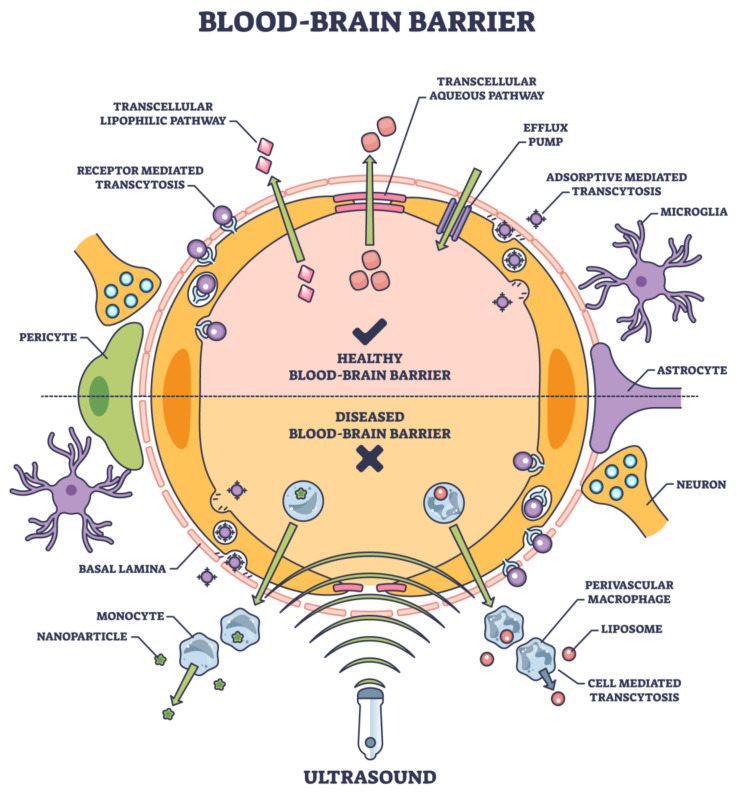
Noninvasive tools and their entry into BBB are based on the physical properties, composition, surface chemistry, and target ligands. [Adobe Stock Photo_603623040].

The BBB poses a monumental challenge for reaching therapeutic agents to the central CNS in the grand drug delivery arena. The development of effective, non-invasive, device-mediated techniques for drug delivery across the BBB has been fraught with challenges but illuminated by moments of innovation and breakthrough. As the field progresses, it is imperative to prioritize safety, ensuring that the integrity and function of the BBB are not compromised in the long term. With continued research and interdisciplinary collaboration, the dream of effective and patient-friendly CNS drug delivery methods may soon become a reality (Figure 4).

The drug’s solubility, surface charge distribution, molecular weight, particle size, and other factors should all be considered while creating the formulation [76].

While often successful in peripheral tissues, traditional pharmacological strategies have encountered substantial limitations when aiming for the brain. These methods aim to increase BBB permeability transiently and safely, allowing for targeted drug delivery without the drawbacks of the traditional methods [77]. Many small molecules and virtually all large molecule biologics, including therapeutic proteins, RNA therapeutics, and antibodies, face impediments in crossing the BBB [9]. Figure 4 lists several approaches to noninvasive deliveries.

Transcellular (drug delivery via cells) and paracellular (drug delivery between adjacent cells) are the two main routes that frequently carry drugs to the brain [78]. Ions and solutes passively diffuse across the BBB via the paracellular pathway, which concentration gradients facilitate. Transcellular delivery across the BBB involves the passage of drugs or molecules through endothelial cells, which form the protective barrier for the brain. Unlike paracellular transport, which consists of passing between cells, transcellular transport goes through the cells themselves, making it a critical route for delivering therapeutic agents to the brain. This process encompasses several mechanisms, including passive diffusion of small, lipophilic molecules; carrier-mediated transport facilitated by specific proteins; receptor-mediated transcytosis, where ligands bind to receptors; and adsorptive-mediated transcytosis, where cationic molecules interact with cell surface components. Each mechanism has unique properties and requirements, making them essential in designing drug-delivery strategies for neurological disorders and brain-related diseases [79] (Figure 4).

Several mechanistic mechanisms, including transcytosis, carrier-mediated transport, and passive diffusion, are part of the transcellular route. The main mechanisms are adsorptive-mediated transcytosis (AMT) and receptor-mediated transcytosis (RMT). Among the most studied targets for RMT in brain endothelial cells are the transferrin receptor, insulin receptor, and low-density lipoprotein (LDL) receptor [80]. Current advancements in RMT provide ways to get past the blood-brain barrier and achieve more efficient medicine administration to the brain. Brain medication administration also uses other transporters, such as organic anion and cation transporters, in addition to these mechanical pathways [81].

Several techniques have been developed over the years, endeavoring to overcome the formidable barrier of the BBB and deliver therapeutic agents to the CNS. Conventional methods range from direct injection into the CNS to modifying drug molecules for enhanced permeability. While some of these techniques have had moderate success, they often come with significant drawbacks, such as invasiveness, limited targeting, or potential side effects.

### 6.1. Direct Injections

Techniques like intracerebroventricular (ICV) and intraparenchymal injections bypass the BBB by delivering drugs directly into the brain or cerebrospinal fluid. While effective, these methods are highly invasive, bear risks of infections, and might distribute drugs unevenly [82]. Methods such as intracerebral injections or intraventricular infusions were employed to achieve CNS drug delivery [83]. While these approaches enable a direct route of administration, they are invasive and carry associated risks, including infection, hemorrhage, and potential damage to brain tissue [84]. Moreover, these methods can lead to non-uniform distribution in the brain, potentially yielding areas of over-concentration or insufficient drug coverage [85]. Further complicating the drug delivery scenario is the realization that many CNS disorders, such as AD, PD, and MS, involve multiple brain regions [86]. Targeting these dispersed areas necessitates a systemic approach to drug delivery, ensuring that the therapeutic agent is distributed throughout the brain.

### 6.2. Molecular Trojan Horses

This ingenious method involves coupling therapeutic agents to molecules crossing the BBB via receptor-mediated transcytosis. By “piggybacking” on these molecules, drugs can be sneaked into the brain. While promising, the complexity of this method and potential immunogenic reactions are challenges that need addressing [87]. It is possible to facilitate the entry of chemical drugs into the brain by employing naturally occurring or artificially modified chemicals and certain simple life forms, predominantly viruses, which can traverse the BBB. This drug delivery strategy is usually called the “Trojan horse” approach. Neurotropic viruses are a class of viruses that exhibit a distinct preference for the nervous system and possess the ability to invade neural cells. These viral agents can traverse the BBB and get access to the CNS. Hence, utilizing neurotropic viruses for drug encapsulation and BBB traversal is a highly effective and practical strategy. Adeno-associated virus (AAV) is the prevailing neurotropic viral vector employed in treating neurological illnesses [88].

### 6.3. Biochemical BBB Disruption

Specific agents, like mannitol, can temporarily disrupt the BBB by shrinking endothelial cells. While this allows drugs to enter the CNS, it is a non-specific method that might allow harmful substances to infiltrate the brain, potentially causing side effects [89]. The hyperosmolar technique is employed to transiently disrupt the BBB by generating alterations in osmolarity inside the brain tissue. Usually, an intravenous or intra-arterial infusion of a high-osmolarity solution, predominantly mannitol, facilitates water movement from brain tissue to the blood arteries via osmosis.

Applying mechanical force on the endothelial cells induces mechanical stress, resulting in a transient disturbance of tight junctions. During this phase, the BBB undergoes temporary permeability, facilitating enhanced medication delivery into the brain and enabling therapeutic effects on NDs. Empirical evidence from clinical trials has demonstrated that administering hyperosmolar mannitol through intra-arterial infusion after a BBB breach is a reliable and secure approach for managing central CNS malignancies. The findings from subsequent research using rats indicated that proteomics alterations reverted to their original levels after 96 h. This suggests that the approach employed to induce BBB opening is transient and may be reversed [90]. Nevertheless, it is crucial to acknowledge that the unguided application of hyperosmolar mannitol to open the BBB is an invasive procedure, and its safety merits thorough deliberation.

### 6.4. Nanoparticle-Mediated Delivery

Nanoparticle systems encompass various carriers, including liposomes, polymeric nanoparticles, and solid lipid nanoparticles (SLNP). These carriers are commonly categorized based on their size (usually ranging from 10 to 300 nm in diameter), chemical composition, and physical morphology. Numerous investigations have examined the utilization of nanoparticles ranging in size from 50 to 200 nm in the context of stroke, AD, and PD. The initial formulation of nanoparticles for cancer treatment received regulatory approval decades ago. Nevertheless, the current repertoire of licensed nanoparticle-based therapies and technologies remains limited. For instance, the utilization of lipid nanoparticles (LNP) for administering mRNA COVID-19 vaccines has gained approval. However, it is essential to note that there is currently a lack of approved central nervous system (CNS) therapy products.

There is great interest in optimizing the physicochemical characteristics of nanoparticles to govern their track and permeability, which might be of great significance in crossing the BBB [91]. Many nanocarriers with particle sizes ranging from 1 to 100 nm have been developed due to advancements in nanotechnology. Polymeric nanoparticles (PNPs), solid lipid nanoparticles (SLNPs), liposomes, and micelles are examples of these nanocarriers that have been introduced as treatments for various neurological disorders, as illustrated in Figure 1. More recently developed, sophisticated nanocarriers, such as exosomes [92], prodrugs [93], self-assembled micelles [94], dendrimers [95], PNPs [96], and exosomes [92], have shown great potential over previous delivery methods.

Non-targeted nanoparticles have significant limitations in traversing the intact BBB. Nanoparticles can encapsulate drugs and be designed to target specific receptors or transporters on the BBB, enhancing drug delivery. This field has garnered considerable interest, but concerns about long-term effects and potential toxicity linger [97]. Utilizing tailored nanomedicines to improve brain transport by capitalizing on the compromised BBB resulting from brain illnesses, such as neurodevelopmental disorders, presents a promising strategy for medication delivery [98].

Nanoparticles as non-invasive methods for drug delivery across the BBB have gained traction due to their effectiveness and versatility. They can be engineered to enhance the delivery of various therapeutic agents to the brain, overcoming the formidable obstacle presented by the BBB. One mechanism through which nanoparticles facilitate drug delivery is by encapsulating drugs and protecting them from metabolic degradation in the bloodstream, enhancing their half-life and bioavailability. A study by Saraiva et al. (2016) [97] demonstrated the use of multi-functionalized nanoparticles to deliver anti-inflammatory drugs to the brain, showing a reduction in neuroinflammation in a targeted and controlled manner. Additionally, surface modification of nanoparticles has been explored to improve their ability to cross the BBB. Poly (butyl cyanoacrylate) nanoparticles coated with polysorbate 80 can help deliver drugs like dalargin, tubocurarine, and doxorubicin across the BBB effectively [99].

Targeted delivery is another essential aspect of nanoparticle-based drug delivery, including the development of magnetic nanoparticles, which could be guided to the brain using an external magnetic field, enhancing the site-specific delivery of the encapsulated drugs [100]. Due to their small size and customizable properties, Nanoparticles serve as excellent vehicles for drug delivery. Combined with techniques like FUS, they can be directed precisely, allowing for slow and sustained drug release [101].

Research involving the co-delivery of gold nanoparticles and anticancer drugs to glioblastoma cells showcased enhanced cell uptake and increased therapeutic efficiency, owing to the synergistic combination of nanoparticles and FUS [102].

Nanotechnology offers avenues to develop carriers that can transport drugs and respond to external stimuli, such as temperature or magnetic fields, enabling controlled release at target sites [103]. When combined with focused ultrasound, magnetic-responsive nanoparticles demonstrated synchronized drug release upon reaching targeted brain regions, presenting a dual-control mechanism for drug delivery [104]. Using an external magnetic field to guide their targeted delivery, magnetic nanoparticles to transport therapeutic agents across the BBB are also attempted [10].

### 6.5. Focused Ultrasound (FUS) with Microbubbles

FUS is a non-invasive medical device employing ultrasonic waves to concentrate and transmit energy to locations within tissues accurately. The application of this technique exhibits significant promise in augmenting the transportation of pharmaceutical agents via the BBB to enhance their uptake in the brain for therapeutic purposes. This can be achieved by facilitating the permeability of the BBB or by aiding in the controlled breakdown of microbubbles to facilitate the release of pharmaceuticals [105]). Presently, focused ultrasound (FUS) has been utilized in neurological disorders (NDs) such as AD and PD (PD). This technique can potentially improve the efficacy of brain drug delivery for a wide range of therapeutic agents, including antibodies, nanoparticles, therapeutic viruses, and stem cells. This is achieved through the temporary opening of the BBB. Several studies have investigated the application of FUS in this context [106,107,108,109,110,111]. The combination of FUS and viral vector gene therapy enhances drug transport efficacy to the brain in the context of PD in animal models. The feasibility and safety of FUS-mediated BBB opening of the striatum have been established in clinical surgical operations for PD (PD) [112].

FUS, combined with microbubbles, has emerged as a frontrunner in non-invasive BBB modulation. The process involves injecting microbubbles intravenously and then applying targeted ultrasound waves. The interaction between the microbubbles and the ultrasound waves temporarily increases the permeability of the BBB, allowing for targeted drug delivery. Preclinical studies have shown successful delivery of therapeutic agents, ranging from small molecules to larger biologics, into the brain with this method [113]. The precision of FUS ensures targeted delivery, minimizing potential systemic side effects. One such method, which has garnered significant attention, is focused ultrasound (FUS) in conjunction with microbubbles [114]. The technique involves the transient disruption of the BBB using ultrasound waves targeted to specific brain regions, enabling the delivery of therapeutic agents precisely where needed. Early results from preclinical studies have shown this technique to be both practical and safe, with the BBB being restored within hours post-treatment [115].

### 6.6. Magnetic Resonance Imaging (MRI)

To tackle the invasive nature of the hyperosmolar process, researchers have devised an MRI technique that relies on unenhanced chemical exchange saturation transfer to identify the buildup of mannitol in the intracranial region after the opening of the BBB. This technique holds promise as a prompt imaging tool for optimizing the administration of mannitol-based BBB opening, thereby enhancing its safety and effectiveness [116]. Moreover, implementing hyperosmolar BBB opening techniques in murine models using MRI guidance can effectively mitigate the limitations of inconsistent reproducibility and heterogeneous experimental results commonly observed with the intra-arterial administration of hyperosmolar mannitol [117]. Hence, the exclusive utilization of hyperosmolar mannitol infusion has potential hazards in therapeutic contexts. Nevertheless, integrating MRI guidance improves the safety and effectiveness of osmotic-based BBB opening, thus augmenting its therapeutic significance.

MRI guidance has significantly improved the precision of focused ultrasound techniques. Through MRgFUS, clinicians can visualize the targeted area in real-time, ensuring therapeutic agents’ accurate and effective delivery while monitoring potential complications [118]. A clinical trial exploring the efficacy of MRgFUS for essential tremor treatments showcased the ability to target the thalamus accurately. Patients exhibited substantial improvement in hand tremors, underlining the potential of imaging-guided interventions [119]. In a rat model of ischemic stroke, FUS, combined with microbubbles, facilitated the targeted delivery of neuroprotective drugs. The treated rats exhibited reduced infarct sizes and improved neurological outcomes compared to the control group [120]. For example, trials using FUS and a combination of FUS+MRI for AD trials saw the BBB in the hippocampus and entorhinal cortex open reversibly without adverse effects [121], and patients showed no adverse events and no cognitive or neurological deterioration [122]. The PD trials of 5–7 patients involved the BBB duplication at the parietal–occipital–temporal junction opened reversibly in four patients without side effects [123]. FUS-mediated striatal BBB opening is feasible and safe. In the MPS-II trial of 28 patients, positive changes occurred in 21 patients treated with transferrin receptor ligand, some with mild or moderate, transient, and manageable adverse drug events [124].

### 6.7. Electromagnetic Field Modulation

While relatively nascent, using electromagnetic fields (EMFs) to modulate BBB permeability is gaining traction. EMFs can influence ion channels and transport mechanisms in the endothelial cells of the BBB, leading to transient permeability increases [125]. Preliminary studies show promise, but the exact parameters for effective and safe application and long-term implications remain under investigation [126]. Another intriguing approach is the use of electromagnetic fields. By leveraging the inherent electrical properties of the BBB, researchers are exploring ways to transiently increase its permeability, allowing for the passive diffusion of therapeutic agents into the CNS [127].

Preliminary studies have indicated a potential for this technique, although its long-term effects and safety profile are still under investigation. Emerging techniques promise selectivity, control, and reversibility. For instance, focused ultrasound, when coupled with microbubbles, can be directed at specific brain regions to enhance BBB permeability temporarily. Studies have shown that this technique can deliver a variety of therapeutic agents, including antibodies, to targeted brain areas with minimal side effects. Furthermore, although a relatively new entrant in this domain, electromagnetic fields have demonstrated potential in modulating BBB permeability. Initial studies suggest that such fields can influence molecular transport across the BBB, although the precise mechanisms and long-term safety still require thorough investigation.

### 6.8. Vasoactive Chemicals

Vasoactive chemicals, including CNS ones, can modulate vascular tone and permeability. Several vasoactive chemicals have been investigated to assess their capacity to induce the opening of the BBB and facilitate the transportation of therapeutic medicines into the brain. The chemicals encompass adenosine, bradykinin, histamine, and peptides derived from bee venom. Adenosine, a nucleoside found in nature, has a role in multiple physiological processes, such as regulating blood flow and inflammation [128].

Numerous studies have demonstrated that it can enhance BBB permeability through various mechanisms. For instance, it can activate adenosine receptors on endothelial cells, initiating intracellular signaling pathways that influence the tight junctions between them. These tight junctions play a critical role in maintaining the integrity of the BBB. The process of fibrinolysis can lead to the production of bradykinin through the action of fibrinolytic agents [129]. This bradykinin can then activate bradykinin B2 receptors, resulting in the opening of the BBB. Researchers have employed the BBB opening mechanism to create bradykinin analogs that can improve the transportation of nanocarriers across the BBB to treat glioblastoma [130].

Furthermore, studies have provided evidence that the disruption of the BBB, facilitated by the bradykinin B2 receptor agonist NG291, is confined to a specific area, dependent on the dosage administered, and can be reversed [131]. Histamine, a neurotransmitter, has been observed to potentially facilitate the opening of the BBB [132,133]. However, the precise mechanism by which this occurs remains uncertain.

Recently, there has been a development in the utilization of bee venom peptides as substances to induce the opening of the BBB. It has been demonstrated that these substances can induce reversible opening of the BBB within 24 h when administered at adequate dosages. Doubtless, vasoactive medicines possess significant promise in facilitating the opening of the BBB and enhancing drug transportation to the brain. Nevertheless, the systemic administration of these medications presents uncertain implications for the overall treatment efficacy, as they cannot selectively target the BBB. Enhancing the precision of BBB targeting with pertinent technology would contribute to advancing future clinical studies.

### 6.9. Gut Microbiome [134]

The enhanced longevity of the human life span can be attributed to advancements in diet and healthcare, which the progress of the economy and technology has facilitated. Many microorganisms, encompassing bacteria, archaea, viruses, and diverse eukaryotes, including fungi and protozoa, inhabit distinct ecological niches within the gastrointestinal tract. The term “gut microbiota” is commonly used to refer to this group of microorganisms.

The gut microbiota significantly impacts various areas of human physiology, encompassing nutritional metabolism, anti-infection mechanisms, immune system functionality, and neuron development. The gut microbiota is facing a changing environment due to rapid industrialization, urbanization, and advancements in food and medical technologies. Factors such as increased fast-food consumption have made the gut microbiota more susceptible to vulnerability than previous conditions. Recently, there has been a growing recognition of the significance of gut microbiota due to its crucial involvement in neurodevelopmental disorders (NDs) and its influence on the differentiation, maturation, proliferation, and activation of immune cells residing in the central nervous system (CNS). The gut–brain axis (GBA) facilitates reciprocal communication between the gastrointestinal tract and the central nervous system using neurotransmitters and other metabolites [135,136].

The blood-brain barrier (BBB) can restrict the transit of molecules produced from the stomach into the brain, even though it acts as a gateway for the passage of many essential compounds needed for CNS functioning and secretes substances into the blood and brain essential for preserving CNS homeostasis [137]. Microorganism-associated molecular patterns (MAMPs), for instance, are vital to microorganisms’ structural integrity and cellular processes [138]. Inadvertent elevation or reduction of MAMPs can cause acute or chronic inflammation linked to several neurological conditions.

The BBB’s permeability is linked to several microbial compounds, including vitamins, lipopolysaccharides (LPS), trimethylamines (TMAs), and short-chain fatty acids (SCFAs) [139,140,141]. These compounds may boost the immunological and endocrine systems to prevent neuroinflammation or neurodegeneration or act directly on brain neurons through the blood-brain barrier. The central nervous system (CNS), autonomic nervous system (ANS), enteric nervous system (ENS), and hypothalamic–pituitary–adrenal (HPA) axis make up the bidirectional communication network.

Through the vagus nerve, microbiota and the brain can communicate. The CNS can affect the functions and activities of intestinal cells more efficiently, thanks to the synergy of neurological and hormonal signals [65,66]. Furthermore, gut microbiota influences host health by modifying gut cells and preserving intestinal metabolic and immunological balance [142,143,144]. It’s interesting to note that the microbiota also affects the synthesis of hormones and neurotransmitters, including peptide YY, gam-ma-aminobutyric acid (GABA), serotonin (5-HT), adrenaline, noradrenaline, glucagon-like peptide-1, and dopamine, as well as their precursors. These chemicals act on the CNS or ENS either directly through the vagus nerve or indirectly by affecting the circulation [145].

### 6.10. Surface Transporters

While the activation of BBB surface transporters can improve drug transport, it is essential to consider the drawbacks of this approach, including saturation, limited transport capacity, and inadequate targeting compared to receptor-mediated endocytosis. Consequently, the latter mechanism is commonly employed in drug development to enhance the targeting and penetration of drugs across the BBB. Currently, the prevailing receptor proteins within the BBB encompass transferrin receptors, insulin receptors, and low-density lipoprotein receptor-related proteins. These receptor proteins are frequently coupled with therapeutic protein drugs through fusion with their respective ligands, thereby enhancing the efficacy of drug targeting and facilitating the passage across the BBB [146].

### 6.11. Penetrating Peptides

Penetrating peptides refer to concise sequences of peptides that can efficiently penetrate the BBB. BBB-penetrating peptides’ repertoire includes trans-activating transcriptional activator peptides, R8 peptides, angiopep-2, cell-penetrating peptides, and RVG peptides [147]. Using BBB-targeting ligands for transporting pharmaceuticals across the BBB represents a precise and efficient approach to facilitating drug delivery.

### 6.12. Extracellular Vesicles

One approach to enhance the ability of medications to penetrate the brain is by modifying tiny extracellular vesicles’ surface with different peptides that can cross the BBB. This modification facilitates the effective transport of drugs to the brain [148]. Furthermore, integrating extracellular vesicles and intranasal delivery would undeniably assume a crucial function in managing neurodegenerative disorders (NDs). Extracellular vesicles serve as inherent vehicles for drug delivery and possess the ability to traverse the BBB readily. Consequently, it is common practice among researchers to employ extracellular vesicles as carriers for drug encapsulation, aiming to enhance the transportation of pharmaceuticals across the BBB where the improved permeability of the BBB can be achieved by utilizing tiny extracellular vesicles produced from glial cells, which are loaded with tetraspanin 2 siRNA [149].

### 6.13. Liposomes

Like extracellular vesicles, liposomes are also employed as carriers for drug administration across the BBB. Nevertheless, it is essential to acknowledge that liposomes exclusively exhibit BBB penetration capabilities rather than BBB targeting abilities. Consequently, these compounds are frequently co-administered with BBB-targeting agents to elicit their desired outcomes. It has been demonstrated that including neurotransmitter-derived lipids in lipid nanoparticles (LNPs) that are impervious to the BBB facilitates the ability of LNPs to traverse the BBB [150].

### 6.14. Optical Imaging

Integrating optical imaging with non-invasive techniques offers detailed real-time visualization at a cellular level. Optogenetics allows for the control of neuronal activity using light, suggesting potential therapeutic applications [151]. A study involving the treatment of Parkinson’s symptoms in rodents used optogenetics. The integration of optical imaging ensured targeted light delivery, leading to controlled neuronal activity and symptom alleviation [152]. Precision is critical in non-invasive drug delivery. The advent of novel imaging techniques promises better visualization, improved targeting, and real-time monitoring of drug delivery [153]. The integration of diffuse optical tomography with ultrasound has shown potential in providing real-time imaging of drug deposition and tissue response, enhancing the safety and efficacy of drug delivery [154].

### 6.15. Peptides

Peptides, with their ability to interact with specific BBB receptors, play a pivotal role in promoting receptor-mediated transcytosis, a mechanism by which peptides facilitate the delivery of an array of therapeutic agents like small molecules, proteins, and genes across the BBB. These peptides target endogenous receptor systems, ensuring a selective and efficient transfer [155].

The use of peptides in noninvasively facilitating drug delivery across the BBB has been a subject of intensive research, attributing to their ability to harness endogenous transport mechanisms without compromising the barrier’s structural integrity. One prominent strategy involves the design of peptides that can undergo receptor-mediated transcytosis. These peptides are specifically tailored to bind to specific receptors on the endothelial cells of the BBB, instigating a process that transports them, along with their drug cargo, into the brain. How peptides could be engineered for targeted delivery, enhancing the selectivity and efficiency of drug transport while minimizing systemic exposure, has been reported [156].

Additionally, the use of cell-penetrating peptides (CPPs) has gained attention. CPPs can facilitate the transport of various bioactive cargoes, including small molecules, peptides, proteins, and nucleic acids, across the BBB, particularly the CPP-mediated [157] transport. Furthermore, developing dual-functional peptides that combine BBB targeting and drug delivery is an emerging trend. Constructing a dual-functional peptide that could transport across the BBB and target glioma cells underscores the advancements in precision medicine [158].

### 6.16. Antibodies

Antibodies, especially monoclonal ones, have been significantly instrumental in drug delivery across the BBB. They can uniquely target specific receptors on the endothelial cells lining the BBB, enabling receptor-mediated transcytosis. This process involves binding antibodies to these receptors, initiating an internalization process that transports them and their attached drug cargo across the BBB. The efficacy of antibodies in drug delivery can be enhanced by reducing their affinity for a transcytosis target, consequently boosting their brain uptake [159]. The bispecific antibodies, designed to engage with the transferrin receptor to facilitate BBB crossing and an amyloid-beta peptide to reduce its pathological accumulation in AD, exemplify engineered antibodies’ dual targeting capability and therapeutic potential [160]. Antibody engineering to improve antibodies’ pharmacokinetics increases their potential as drug carriers to the brain [161]. Antibodies are equally instrumental in bypassing the BBB. A specific example is the monoclonal antibody against the transferrin receptor, where the mechanism by which antibodies with reduced affinity for a transcytosis target can significantly enhance the brain’s uptake of therapeutic antibodies [159].

### 6.17. Intranasal

In recent years, intranasal and intrathecal administration have developed viable and effective methods for enhancing brain-targeting efficiency in medication administration. The intranasal route of drug administration is a favorable method for the targeted delivery of medications to the central CNS owing to the abundant vascularization of the nasal cavity, which is situated near the brain. Research findings have indicated that the intranasal administration of exosomes can lead to a notable accumulation of these particles in the brains of animals with PD [162]. The intranasal delivery of dantrolene has demonstrated enhanced brain concentration and prolonged duration of action compared to oral administration [163]. The process of intrathecal administration entails the direct delivery of medications into the cerebrospinal fluid (CSF) that surrounds the brain and spinal cord. This method bypasses the BBB and enables direct drug delivery to the CNS. Nevertheless, intrathecal administration is constrained because of its invasive characteristics, technical intricacy, probable unfavorable responses, restricted indications, and elevated expenses [164]. Capitalizing on the direct connection between the nasal cavity and the brain via the olfactory and trigeminal nerves, this method offers a direct pathway for drug delivery to the CNS. While still in its infancy, this approach has shown significant potential, especially for delivering peptides and other macromolecules that traditionally have difficulty crossing the BBB.

### 6.18. Circadian Rhythm [165]

The circadian rhythms, which regulate several human physiological and behavioral activities, are controlled by endogenous biological clocks coordinated by the suprachiasmatic nucleus. The circadian system significantly impacts various physiological functions, encompassing sleep, alertness, and cognitive ability. The perturbation of circadian homeostasis has harmful implications for human well-being. Neurodegenerative illnesses contain a diverse array of symptoms, with a notable characteristic being the presence of diurnal fluctuations in both frequency and intensity.

Furthermore, these illnesses have been found to alter the equilibrium of the circadian system, resulting in a detrimental impact on symptoms and overall quality of life. Increasing evidence indicates a reciprocal association between circadian homeostasis and neurodegeneration, implying that the proper functioning of circadian rhythms may play a crucial role in advancing neurodegenerative diseases. Hence, the circadian system has emerged as a compelling subject of investigation and a promising avenue for advancements in research and clinical interventions. Investigating the impact of circadian disruption on neurodegenerative disorders can enhance our comprehension of the underlying mechanisms of neurodegeneration and promote the creation of innovative therapies based on circadian rhythms for these debilitating conditions.

The sensitivity of the BBB to medications exhibits variability following the circadian rhythm. It has been demonstrated that the administration of the antiepileptic medicine phenytoin during nighttime has enhanced efficacy in the treatment of seizure models in fruit flies [166]. One potential cost-effective method could involve strategically managing medicine administration time to optimize travel efficiency.

### 6.19. Precision Medicine

Precision medicine encompasses several strategies to achieve more accurate drug targeting, optimal drug dose, refined illness subtyping, and the meticulous management of individual variations. Precise medication targeting and dosage can be accomplished by targeting methodologies, localized drug administration to the specific lesions, and techniques such as co-focused ultrasound in conjunction with microbubbles. Nevertheless, categorizing diseases into subtypes remains ambiguous for neurological disorders (NDs), and comprehending individual variations poses significant difficulties. This signifies a substantial avenue for future therapy of neurodegenerative disorders.

### 6.20. Light-Induced Techniques

Optogenetics and photo biomodulation harness light to affect cellular activity. Recent advancements indicate potential in modulating BBB permeability using specific wavelengths of light, especially when combined with photosensitive agents [167]. While these techniques are in their infancy regarding BBB modulation, the non-invasive nature and advancements in targeted light delivery make them an area of keen interest.

### 6.21. Radiofrequency (RF) Modulation

RF energy, a form of electromagnetic radiation, has been explored for its potential to increase the BBB’s permeability. The concept involves using RF pulses that induce temporary and reversible changes in the BBB, facilitating drug entry [168]. Though the method holds promise, defining the precise parameters for safe and effective delivery is a significant focus of ongoing research.

### 6.22. Thermal Techniques

Mild hyperthermia, induced by devices like microwave applicators, can increase BBB permeability. The technique exploits the sensitivity of BBB endothelial cells to temperature changes, allowing for a temporary “opening” of the barrier [169]. While the method is promising, ensuring precise temperature control and preventing potential thermal damage to surrounding tissues remain challenges.

These non-invasive, device-mediated techniques significantly depart from traditional methods, favoring precision, control, and safety. The advancements promise more effective drug delivery to the CNS and open avenues for delivering a broader range of therapeutic agents, including those previously deemed unsuitable due to their inability to cross the BBB [170]. As research progresses, there is optimism that these techniques will pave the way for novel treatments, offering hope to millions affected by neurological diseases.

## 7. Ex Vivo Modeling

Building in vivo and in vitro BBB models and the innovation of research methods are crucial for several aspects of BBB research, including restoring BBB integrity and enhancing drug penetration efficiency across the BBB. Hence, this article concisely overviews research models such as BBB animal, organoid models, and BBB chips. Additionally, it briefly introduces several invasive and non-invasive BBB research methods, serving as a valuable resource for fellow researchers.

### 7.1. Animal Modes

The utilization of zebrafish and Drosophila models is prevalent in BBB research due to its numerous advantages. These advantages encompass a fast generation time, cost-effectiveness, advanced genetic manipulation capabilities, and the ability to analyze intricate behaviors. Utilizing the zebrafish model provides an added benefit due to the transparency of its early-stage embryos, enabling direct visualization of the BBB.

### 7.2. Organoid Model

The BBB organoid model, is a scientific approach used to study the BBB in a laboratory setting. The construction of in vitro organoid models of the BBB involves culturing and combining different components that make up the BBB to mimic its functional properties observed in living animals, considering the established composition and structure of the BBB. Brain microvascular endothelial cells (BMECs) represent a crucial cellular component within the BBB. The creation of an in vitro organoid model with partial BBB functionality can be achieved by co-culturing brain microvascular endothelial cells (BMECs) with other cell types of the BBB, such as astrocytes and pericytes, on a permeable membrane.

### 7.3. The BBB Chip

The BBB chip is a microfluidic device designed to replicate the human BBB in an in vitro setting. The primary objective of this study is to investigate the intricate dynamics between pharmaceutical substances and various small molecules concerning the BBB, with a particular focus on assessing their capacity to permeate this physiological barrier. Compared to conventional in vitro methods and animal models utilized in BBB research, the BBB chip offers a more authentic in vitro BBB model, diminishes the necessity for animal experimentation, expedites the drug development trajectory, and facilitates the advancement of tailored therapeutic interventions for neurological disorders [171,172,173].

The development of BBB chips produced from human induced pluripotent stem cells (iPSCs) has been achieved by researchers. These chips demonstrate physiologically realistic trans-endothelial resistance and effectively predict the blood–brain permeability of pharmacokinetic substances.

## 8. Summary

The appeal of non-invasive device-mediated techniques lies in their ability to target specific brain regions while circumventing traditional drug delivery barriers. However, as with any emerging technology, they come with advantages, challenges, and future possibilities that need addressing.

### 8.1. Advantages

- Precision and Specificity: Techniques like FUS offer pinpoint accuracy in targeting specific brain regions [174]. This ensures that only the desired area is treated, reducing the risk of systemic side effects.
- Versatility: The non-invasive nature of these techniques makes them suitable for a wide range of applications, from delivering small-molecule drugs to larger molecules like antibodies or even genes.
- Minimally Disruptive: Unlike invasive methods, which can cause tissue damage or infection, non-invasive techniques are generally safer with minimal post-procedure complications.
- Repeatability: Given their non-destructive nature, these techniques can be applied repeatedly, allowing for chronic treatments or adjustments [175].

### 8.2. Challenges

- Understanding Long-term Effects: While initial studies are promising, the long-term effects of repeated BBB disruption or electromagnetic field exposure remain to be comprehensively understood [176].
- Optimization of Parameters: Each technique requires fine-tuning parameters to ensure efficacy without compromising safety. For instance, ultrasound’s right frequency and duration of the optimal wavelength for light-induced techniques are vital for success [177].
- Systemic Side Effects: Despite targeted delivery, there is a potential for drugs to diffuse from the target site, leading to unintended effects elsewhere in the brain or body.
- Technological Limitations: Current devices may not be optimized for deep brain structures or use in specific populations like children or older adults [178].

## 9. Future Perspectives

- Combination Therapies: Combining non-invasive techniques could further improve delivery efficacy. For instance, using FUS to enhance nanoparticle delivery across the BBB could combine the strengths of both methods [179].
- Advanced Monitoring: Integrating real-time imaging, like MRI, with drug delivery can provide immediate feedback, ensuring optimal delivery and minimizing potential risks [180].
- Personalized Approaches: As our understanding grows, it may be possible to tailor techniques to individual patients based on their unique anatomy, pathology, and therapeutic needs [181].
- Expansion to Other Diseases: While the current focus might be on neurological disorders, the potential exists to expand these techniques for other conditions, from brain tumors to systemic illnesses with CNS involvement [182].

### Applications

The potential clinical applications of non-invasive device-mediated techniques are vast. As more research unravels their potential and addresses the associated challenges, there is hope these techniques will transform the landscape of neurological treatment, ushering in an era of more effective and less invasive therapeutic interventions. The progress of non-invasive device-mediated techniques is closely intertwined with technological advancements and the integration of imaging modalities. These dual advancements allow for real-time monitoring and adjustment, ensuring safety and efficacy during treatments. The synergy between technological advances, integration with imaging modalities, and the introduction of computational methods heralds a new era for non-invasive device-mediated drug delivery. These integrative approaches promise enhanced efficacy and pave the way for personalized treatments tailored to individual patient needs.

The evolving landscape of non-invasive device-mediated drug delivery presents both challenges and opportunities. Addressing current limitations will determine the trajectory of this field in the coming years as more conceptual and practical inquiries into the science and the art of overcoming the hurdle of BBB to treat diseases become evident.

Combining non-invasive device-mediated delivery with emerging therapies like gene editing or stem cell therapies can potentiate therapeutic outcomes. Accurately delivering these agents to targeted areas can amplify their efficacy [183]. A recent study employed focused ultrasound to facilitate the delivery of CRISPR/Cas9 components to the brain, showcasing potential applications in genetic disorders [184].

## 10. Conclusions

BBB offers a promising frontier in neurotherapeutics. While they bring many advantages, challenges that must be addressed through rigorous research persist. The future, replete with possibilities, could see these techniques revolutionizing not just neuroscience but the broader landscape of medicine.

The promising advantages and ongoing developments in non-invasive device-mediated techniques have paved the way for potential clinical applications, ranging from NDs to brain tumors.

The field of non-invasive device-mediated drug delivery stands at an exciting juncture. The convergence of technological advancements, biomedical research, and clinical needs promises to revolutionize treatment modalities for various diseases, particularly those affecting the brain. Addressing current challenges and capitalizing on emerging opportunities will be pivotal. With continued interdisciplinary collaboration, investment, and innovation, the full potential of these techniques can be realized, heralding a new era in medical treatments.

As we gaze into the horizon of non-invasive device-mediated drug delivery, a range of innovations and advancements come into view. These innovations, stemming from diverse areas of science and engineering, have the potential to address existing challenges and propel the field into novel therapeutic paradigms.

The potential of non-invasive device-mediated drug delivery is vast, and as technology and biomedical research continue to evolve together, new avenues and possibilities emerge. The intersection of these advancements holds immense promise for transforming the therapeutic landscape. As research progresses and innovations are integrated into clinical practice, patients worldwide stand to benefit from more effective, targeted, and safer treatments.

While the journey of non-invasive device-mediated drug delivery is intertwined with challenges and regulatory intricacies, their promise and potential are undeniably transformative. Here is a closer look at what the horizon might hold.

The realm of non-invasive device-mediated drug delivery is on the cusp of redefining therapeutic interventions, especially for conditions previously deemed untreatable. The intertwined dance of science, technology, ethics, and humanity promises a future where treatments are effective and compassionate. As we stride ahead, let this journey be marked by innovation, inclusivity, and an unwavering commitment to enhancing human lives.

Harnessing the power of artificial intelligence (AI) and machine learning can optimize treatment parameters, predict patient responses, and improve therapeutic outcomes [185]. A recent project employed AI algorithms to analyze patient data and optimize focused ultrasound settings, enhancing treatment precision and reducing side effects [186].
